# Supplementary figures and images for: Need of care in interpreting Google Trends-based COVID-19 infodemiological study results: potential risk of false-positivity
Source: BMC Med Res Methodol. 2021 Jul 18;21:147. doi: 10.1186/s12874-021-01338-2 (PMC8286439; doi:10.1186/s12874-021-01338-2)

**AU**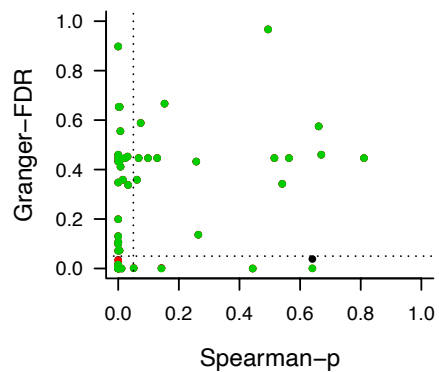**CA**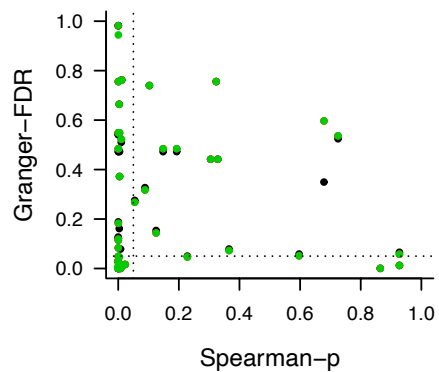**GB**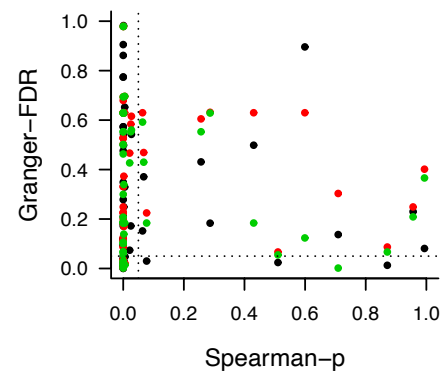**IE**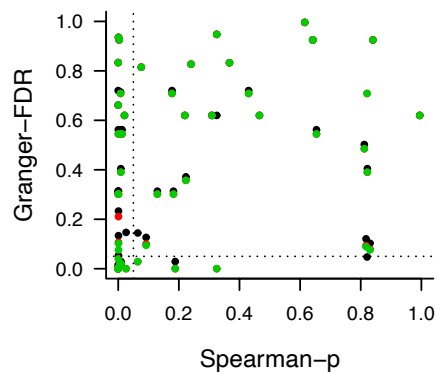**IN**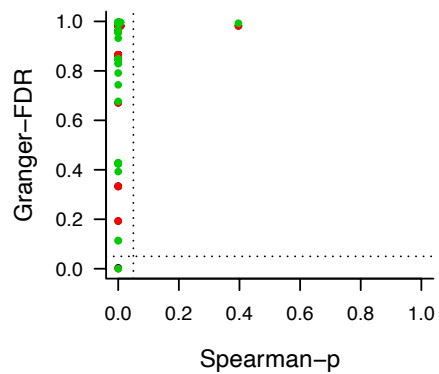**JP**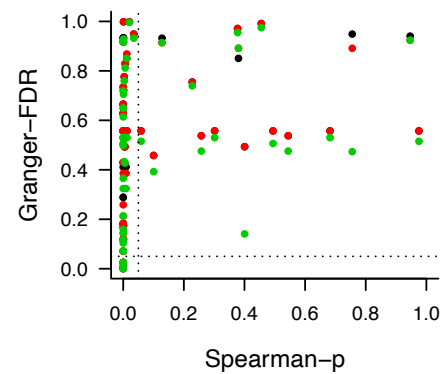

• Lag=4  
• Lag=6  
• Lag=8

**SG**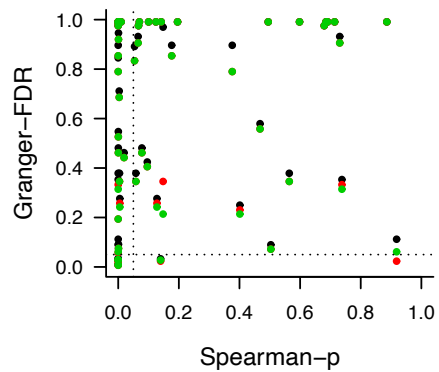**US**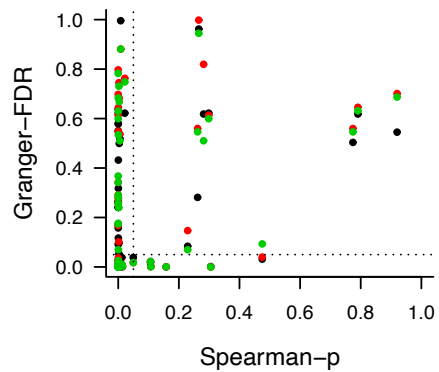**ZA**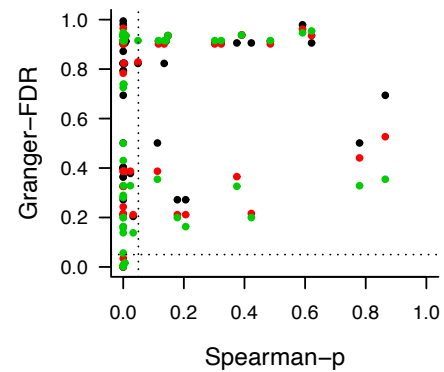

Supplement: Supplementary file 2 — Additional file 2. Difference in the distribution between Spearman’s rank correlation p-value and Granger causality FDR. In each country from upper left to lower right in an alphabetical order, each dot plots the Spearman’s rank correlation p-value and the Granger-causality FDR value (each of lag 4, 6, and 8: differently colored) of the same search keyword. Vertical and horizontal dotted lines show value of 0.05 for the reference of significance. For many of the examined keywords, the p-value/FDR clearly became non-significant level when using Granger-causality analysis (in y-axis) instead of Spearman’s rank correlation test (in x-axis). Abbreviations: AU, Australia; CA, Canada; GB, Great Britain; IE, Ireland; IN, India; JP, Japan; SG, Singapore; US, United States; ZA, South Africa; FDR, false discovery rate. [file 12874_2021_1338_MOESM2_ESM.pdf]
